# Exploring human factors in the operating room: scoping review of training offerings for healthcare professionals

**DOI:** 10.1093/bjsopen/zrac011

**Published:** 2022-03-29

**Authors:** Alex Lee, Alexandra Finstad, Ben Tipney, Tyler Lamb, Alvi Rahman, Kirsten Devenny, Jad Abou Khalil, Craig Kuziemsky, Fady Balaa

**Affiliations:** Faculty of Medicine, University of Ottawa, Ottawa, ON, Canada; Faculty of Medicine, University of Ottawa, Ottawa, ON, Canada; MedLed Ltd, Slough, UK; Division of General Surgery, Department of Surgery, University of Ottawa, Ottawa, ON, Canada; Department of Epidemiology, Biostatistics and Occupational Health, McGill University, Montreal, QC, Canada; Saegis, Canadian Medical Protective Association, Ottawa, ON, Canada; Division of General Surgery, Department of Surgery, University of Ottawa, Ottawa, ON, Canada; Office of Research Services and School of Business, MacEwan University, AB, Canada; Division of General Surgery, Department of Surgery, University of Ottawa, Ottawa, ON, Canada

## Abstract

**Background:**

Human factors (HF) integration can improve patient safety in the operating room (OR), but the depth of current knowledge remains unknown. This study aimed to explore the content of HF training for the operative environment.

**Methods:**

We searched six bibliographic databases for studies describing HF interventions for the OR. Skills taught were classified using the Chartered Institute of Ergonomics and Human Factors (CIEHF) framework, consisting of 67 knowledge areas belonging to five categories: psychology; people and systems; methods and tools; anatomy and physiology; and work environment.

**Results:**

Of 1851 results, 28 studies were included, representing 27 unique interventions. HF training was mostly delivered to interdisciplinary groups (*n* = 19; 70 per cent) of surgeons (*n* = 16; 59 per cent), nurses (*n* = 15; 56 per cent), and postgraduate surgical trainees (*n* = 11; 41 per cent). Interactive methods (multimedia, simulation) were used for teaching in all studies. Of the CIEHF knowledge areas, all 27 interventions taught ‘behaviours and attitudes’ (psychology) and ‘team work’ (people and systems). Other skills included ‘communication’ (*n* = 25; 93 per cent), ‘situation awareness’ (*n* = 23; 85 per cent), and ‘leadership’ (*n* = 20; 74 per cent). Anatomy and physiology were taught by one intervention, while none taught knowledge areas under work environment.

**Conclusion:**

Expanding HF education requires a broader inclusion of the entirety of sociotechnical factors such as contributions of the work environment, technology, and broader organizational culture on OR safety to a wider range of stakeholders.

## Teaser

To examine the scope of application of human factors for the operating room, this study explored the content of emphasis in human factors education to date. We found that human factors training is limited to teaching behavioural, non-technical skills but may not address the entirety of sociotechnical factors that impact operating room safety. There is room to expand the range and scope of human factors for the operating room in future curricula to include contributions of the work environment, technology, and broader organizational culture on safety to a wider range of stakeholders.

## Introduction

The operating room (OR) is a unique and complex intersection between multiple personnel (e.g. surgeons, anaesthesiologists, nurses, and other perioperative workers), various equipment and tools (e.g. surgical devices and monitors), and the workplace (e.g. OR access, staff availability, and operational costs). Consequently, the unpredictable and critical nature of the intraoperative setting can be responsible for up to 74.9 per cent of incidents that occur in patients admitted for surgical care^1^. Surgical safety incidents have traditionally been blamed on skill deficiencies in the individual clinician. However, it is now accepted that critical events are strongly influenced by the environment in which they operate^2,3^.

The study of human factors (HF) has been implemented to address the entirety of sociotechnical factors that affect process and safety within the OR^4–6^. Historically, HF draws knowledge from other high-risk disciplines, including aviation and military, and has been progressively adapted to the OR to optimize performance and system efficiency through, for example, crew resource management (CRM) training and safety checklists^4,5,7,8^. The intersection of numerous fields, including psychology and technology, has probably led to a considerable variation in the terminology, concept, and application of HF^9–11^, resulting in a heterogeneous awareness around this topic^10,12,13^.

This complexity introduces unique challenges to transform ORs into high-reliability environments, seeking to optimize the quality of care, patient safety, and costs^6,14,15^. Effective and meaningful HF integration in the OR may ultimately depend on establishing a shared framework delivered through knowledge translation and education among stakeholders^13,16^. To elicit how HF is being understood and applied in the OR, this study aims to explore the content and tools used in HF education and training for the intraoperative environment.

## Methods

This scoping review followed the PRISMA-ScR guidelines^17^. This study was also appraised by key stakeholders, including OR clinicians (J.A.K., F.B.), an HF expert (B.T.), and a health systems research expert (C.K.). A study protocol was developed a priori and published in a peer-reviewed journal^18^.

### Search strategy

Six electronic bibliographic databases, including MEDLINE (Ovid), Embase (Ovid), PsycINFO (Ovid), CINAHL (EBSCOhost), Health and Psychosocial Instruments (Ovid), and ERIC (Ovid), were searched up to August 2020, in consultation with a health sciences research librarian who helped to refine the search strategy. No previous systematic or scoping reviews have explored this topic.

The full search strategy used for MEDLINE is reported in *Table S1*. The search strategy combined both keywords and indexed terms related to ‘human factors’, ‘operating room’, and ‘education’. All references were checked to identify additional missed papers eventually included for screening.

### Eligibility criteria

All studies reporting HF training or education interventions in the operative setting were included according to the PRISMA-ScR criteria of population, concept, and context. The population included healthcare professionals or trainees (e.g. surgeons, anaesthesiologists, or nurses) and non-clinical operating room personnel (e.g. OR administrators, housekeeping staff, and hospital porters). The concept included any individual educational or training intervention labelled ‘human factors’ for the OR setting. The context consisted of original research articles published in English, including single and double-arm studies, qualitative and quantitative studies, randomized controlled trials, and quasi-experimental studies. Studies not reporting original data (e.g. editorials and commentaries) or the content of the HF training, and conference abstracts were excluded.

### Study selection

The titles and abstracts of the retrieved studies were independently screened by two reviewers (A.L. and A.F.), who evaluated the full-text articles of potentially eligible studies for inclusion. Reasons for exclusion were documented and summarized. Any disagreements between the two reviewers were resolved by consensus or, if necessary, by a third reviewer (F.B.).

### Data charting

Data from the included articles were charted in a standardized data spreadsheet using Microsoft Excel version 16.46, which the authors calibrated prior to the search. Charted data included the study characteristics (authors, year of publication, country of study, indexed keywords, research type); training participants (number, type, and level of training of learners and instructors, interdisciplinary *versus* intradisciplinary learning group); training design (training developers, type of teaching methods or tools used, duration and frequency of training, learner assessment tool used); and training content (skills or concepts taught, quantitative or qualitative outcomes measured and reported, feedback from participants). When HF was a component of broad interventions, only HF data were charted.

### Data synthesis and summary of results

A meta-analysis and a formal methodological quality assessment were not performed owing to the heterogeneity of the included studies. Charted data were summarized in tables or diagrams, with a narrative summary to show and explore the spectrum of HF training for the operative setting. To further examine HF-labelled teaching interventions for the operative setting, the training content was assessed according to the Chartered Institute of Ergonomics and Human Factors (CIEHF; *Table S2*), which includes 67 HF knowledge areas divided into five main categories: anatomy and physiology; psychology; people and systems; work environment; and methods and tools^19^. Any skills or concepts deemed not captured by the CIEHF knowledge areas were also recorded. Inter-rater classification reliability was assessed using Cohen’s kappa statistic. An assessment of the quality of evidence on the topic of interest of each study was performed using the Medical Education Research Study Quality Instrument (MERSQI)^20^. With a maximum score of 18, higher total MERSQI scores have shown to be associated with better expert quality ratings, 3-year citation rate, journal impact factor, and funding amount for the intervention^21^.

## Results

### Search results

The search yielded a total of 1851 studies, of which 112 were appropriate for full-text assessment. A total of 28 studies met the eligibility criteria and were included in this scoping review. The PRISMA flow chart is shown in *Fig. S1*.

### Characteristics of the including studies

The included studies were published between 1996 and 2019, with 61 per cent of the articles published since 2010 (*Table S3*). Of the 28 eligible studies, two evaluated the same intervention over different time periods^22,23^, for a total of 27 single training offerings. Most interventions were from the UK (*n* = 13; 48 per cent), the USA (*n* = 5; 19 per cent), and Australia (*n* = 2; 7 per cent). Three of the 27 interventions were developed by the same research group in the UK^24–26^. Common indexed keywords reported by different studies included ‘safety’ (*n* = 7; 26 per cent), ‘teamwork’ (*n* = 5; 19 per cent), ‘simulation’ (*n* = 4; 15 per cent), and ‘nontechnical skills’ (*n* = 3; 11 per cent). In 24 studies, the primary objective was to describe or evaluate the HF training intervention. Of the remaining studies, one assessed behavioural marker systems in the context of HF training^27^, and two assessed both the training offering and the behavioural marker system or the HF evaluation method^28,29^. A total of 23 studies had quantitative data appropriate for MERSQI assessment (*Table S3*). The mean score was 11.7/18 (range 8.5 to 14.5).

### Training population and methods

**Fig. 1:**
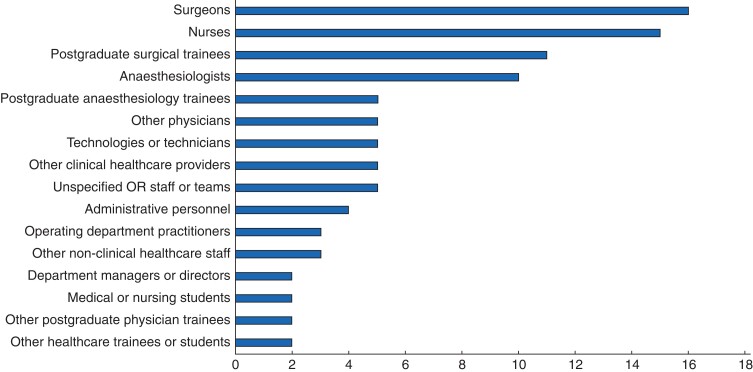
Types and prevalence of learners in human factors training interventions Other clinical healthcare providers included respiratory therapists, dieticians, and physician assistants. Other non-clinical healthcare staff included porters, housekeeping workers, and orderlies. OR, operating room.

HF training was most often delivered to interdisciplinary (*n* = 19; 70 per cent), rather than intradisciplinary (*n* = 8; 30 per cent), groups of learners, especially surgeons (*n* = 16; 59 per cent), nurses (*n* = 15; 56 per cent), and postgraduate surgical trainees (*n* = 11; 41 per cent) (*Fig. 1*). In contrast, non-clinical staff (*n* = 3; 11 per cent) and administrative personnel (*n* = 4; 15 per cent) were included in fewer studies.

HF content was taught and/or evaluated by trainers with variable expertise, including HF-trained clinical faculty members or CRM experts (*Table 1*). Of note, eight training offers involved an instructor’s course with a ‘train-the-trainer’ approach^28,30–36^. The number of learners and trainers varied widely across the studies.

**Table 1:** Human factors training: participants and design

Study	Training method	Type of learner	No. of learners	Type of trainer/evaluator	Training duration
**Ansari *et al*., 2020**	Classroom, activities, social media, forum theatre, behavioural simulations, *in situ* simulation with debriefing	Midwives, theatre staff, midwifery care assistants, neonatologists, anaesthetists, obstetricians	Total of 269 participants: 152 midwives, 38 obstetricians, 20 theatre staff, 17 midwifery care assistants, 27 neonatologists, 15 anaesthetists	Attainability (experts in civil/military aviation) trained staff (midwives, obstetricians, theatre staff, midwifery care assistants, neonatologists, anaesthetists) to become trainers	6-month study period 2-day ‘train-the-trainer’ course 15–20 min of *in situ* simulation
**Stewart-Parker *et al*., 2017**	Simulation with simulator and debriefing, lectures, multimedia presentations, case studies, interactive team-working exercises	Scrub nurses, operating department practitioners, surgical technologists, healthcare assistants, core surgical and anaesthesia trainees (excluded newly qualified physicians)	Total of 68 participants: 26 core surgical trainees, 25 scrub nurses, 10 operating department practitioners, 4 healthcare assistants, 3 anaesthesia trainees	Senior nurses, consultants, registrars, core trainees	1 day
**Mancuso *et al*., 2016**	Lectures, videos, small-group breakout sessions, role modelling, feedback by trainers	All members of obstetric and neonatal teams involved in caesarean births: physicians, fellows, residents, nurses, respiratory therapists, midwives, technicians, physician assistants, department directors, and managers	Total of 367 participants	Trainers from Safer Healthcare Role modelling by obstetrics and neonatal medical directors and nurse practitioners who completed CRM training and resuscitation team training	5-month training period Total of 12 CRM training sessions
**Saleh *et al*., 2016**	Simulation with actors, debriefing with video playback	Ophthalmologists (trainee to attending level), nurses	Total of 20 participants	Experienced/senior ophthalmologists, nurses	Unspecified
**Stephens *et al*., 2016**	Core training day: presentation, practical team exercises, workshops (small-group work, facilitated whole-group discussion) Sustainment strategy: theatre newsletters, safety data display, after action review, meetings, seminars	Surgeons (orthopaedics, maxillofacial, renal, vascular, trauma, neurosurgery), anaesthetists, theatre and recovery nurses, radiographers, healthcare support workers, porters, and schedulers (junior to senior level)	10–15 participants per core training day Total of 122 participants: 46 surgeons, 30 anaesthetists, 46 nurses, and other health professionals (theatre and recovery nurses, radiographers, operating department practitioners, porters)	HF and team training facilitators Seminars delivered by safety culture experts	1-day core training day
**Heaton *et al*., 2016**	Lecture, simulation using a simulator in mock setting (OR, outpatient clinic, inpatient ward), debriefing	Orthopaedic residents (postgraduate 5–10 years)	Total of 26 participants, six participants per course	Orthopaedic attendings, senior residents, full-time course facilitators from the Department of Medical Education trained in CRM	Six simulation scenarios, otherwise unspecified
**Tsuburaya *et al*., 2016**	E-learning	Upper gastrointestinal surgeons	Total of six participants	Attending surgeons at the department of gastrointestinal surgery as NOTSS assessors	Unspecified
**Chan *et al*., 2016**	Classroom, games, videos, discussion, exercises	Nurses and doctors (from medicine, surgery, obstetrics and gynaecology, paediatrics, accident and emergency department, ICU, anaesthesiology and operating theatre services, clinical oncology, orthopaedics and traumatology, radiology and imaging, other departments)	Total of 164 participants: 139 nurses, 25 physicians (42 from medicine, 8 from surgery, 13 from obstetrics and gynaecology, 16 from paediatrics, 6 from accident and emergency department, 8 from ICU, 22 from anaesthesiology and operating theatre services, 10 from clinical oncology, 9 from orthopaedics and traumatology, 9 from radiology and imaging, 21 from other departments)	CRM-certified instructors	5 h
**Maertens *et al*., 2016**	Video-based learning, e-learning	Medical students (senior year), vascular surgeons (performed ≥ 100 endovascular procedures)	Total of 49 participants: 29 medical students, 20 vascular surgeons	Endovascular surgeons with educational background	Unspecified
**Timmons *et al*., 2015**	Classroom with lectures, discussion, group exercises, practicals	Faculty group: consultant physicians and surgeons, nurses, theatre practitioners Course participants: emergency department and OR clinicians and nurses (junior to senior level)	Total of 39 participants: 20 faculty groups, 19 course participants	HF experts in aviation who trained the faculty group to train the course participants	6 days
**Jones *et al*., 2014**	Microteaching, lecture, video, interactive group discussion, review of scenarios, simulation using a simulator	Second-year (core surgical training) surgical trainees	Total of 33 participants, 18 participants per course	Faculty staff who underwent an internal programme of development in the delivery of non-technical skills teaching and structured debriefing in simulation training	1 day
**De Korne *et al*., 2014**	Classroom, presentation, discussion, flight simulation, video playback with feedback	Ophthalmologists, anaesthesiologists, internists, residents, surgical nursing, anaesthetic assistants, nursing, outpatient allied health staff, administrative staff	Total of 252 participants: 21 ophthalmologists, 2 anaesthesiologists, 2 internists, 20 residents, 34 surgical nursing, 17 anaesthetic assistants, 35 nursing, 65 outpatient allied health staff, 56 administrative staff	Aviation safety experts trained in CRM	12 h (three 4-h interactive classroom sessions)
**Davies *et al*., 2014**	Pre-reading, interactive exercises, storytelling, reflection on practice, videos	Surgeons, nurses, anaesthetists	Unspecified	Nurses, anaesthetists, surgeons	Unspecified
**Bleakley *et al*., 2006, 2012**	Seminars, small-group discussion, presentations, maintenance with meetings and newsletters	Operating theatre staff	Total of 302 participants in year 1 and 332 in year 2	Human resources management training firm, international experts in non-technical skills, researchers from psychological consultancy firm, theatre staff, a research team	6-month period for introducing intervention, 6-month maintenance period
**Hull *et al*., 2012**	Audiovisual materials (PPT, video clips), didactic teaching (lecture presentations), interactive tasks, small-group activities, group discussion	Postgraduate students in pharmacy, economics, engineering, physiology, epidemiology, optometry, public health, paediatrics, industrial design, psychology, nursing	Total of 17 participants	HF and psychology experts, clinical expert	1 day (or two half-day sessions, 4 h per session)
**Morgan *et al*., 2011**	Simulation, CRM training-guided debriefing using presentation and videotapes of participants’ performance	Practising anaesthetists	Total of 59 participants	Experts in simulation debriefing, video reviewers (anaesthesiologist, anaesthesia assistant)	45-min simulation, 45–60-minute CRM training-guided debriefing
**Catchpole *et al*., 2010**	Classroom with interactive modules, discussion, OR coaching	Surgeons, anaesthetists, and nurses (junior to consultant level)	Unspecified	Aviation trainers experienced HF observers	1–2-day classroom, 8 sessions of OR coaching per site
**Hurlbert and Garrett, 2009**	Preoperative briefing, postoperative briefing, presentation, individual coaching	OR staff, nurses, and surgeons from all major surgical specialties	Total of 260 participants: 200 OR staff, 60 surgeons	Trainers from Safer Healthcare, cardiothoracic surgeon, paediatric surgeon	4 h
**Mason *et al*., 2009**	Course with didactic and interactive sessions	Surgeons from various surgical subspecialties	Total of 16 participants	HF trainer in aviation, clinical psychologist, psychiatrist, consultant surgeon	1 day
**Koutantji *et al*., 2008**	Simulation with the simulator in a virtual operating theatre, presentation, discussion, videotaped simulation operation, classroom roleplay, individual feedback by trainers	Surgeons (registrar), anaesthetists (consultant, registrar), scrub nurses, operating department practitioners	Total of 34 participants (9 teams): 9 surgeons, 9 anaesthetists, 9 scrub nurses, 7 operating department practitioners	Expert observers, psychologists	4–5 h
**Marshall and Manus, 2007**	Classroom, workshop activities, videos, roleplay	Surgeons, nurses, certified RN anaesthetists, technologists, anaesthetists, physician assistants, hospital aides, care partners, unit assistants, clerks, secretaries, administrators, managers, housekeepers, dietitians, others	Total of 688 participants, maximum 35 participants per class	Trainers from Safer Healthcare	4 h
**Undre *et al*., 2007**	Simulation operating theatre with the anaesthetic simulator, discussion, written material	Surgical trainees (senior house officers or registrars), anaesthesia trainees (senior house officers or registrars), nurses (newly qualified to senior scrub nurses), operating department practitioners (newly qualified staff or students)	Total of 80 participants: 20 surgeons, 20 anaesthetists, 20 scrub nurses, 20 operating department practitioners; 4 participants per team	Consultant surgeon, consultant anaesthetist, senior operating theatre nurse, operating department practitioner trainer, project coordinator (trainee surgeon), psychologists	0.5 days
**Moorthy *et al*., 2006**	Simulation in a simulated operating theatre with the anaesthetic simulator	Surgical trainees (junior to senior)	Total of 20 participants, 10 participants per group	HF researcher who provided non-technical feedback Non-technical skills assessment by HF researcher and surgical fellow trained by researchers	Unspecified
**Weller *et al*., 2005**	HF module (one module out of five): course manual, pre-reading, presentation, discussion, games, videos, simulation (simulated crises using simulators), skill stations	Anaesthetists (trainee and specialists)	Unspecified	Trainers who underwent the EMAC Instructors Course External observers/evaluators from Australian and New Zealand College of Anaesthetists	2.5 days
**Grogan *et al*., 2004**	Lectures, case studies with role-playing in simulated scenarios	Nurses, technicians, physicians, and administrative personnel from trauma, emergency department, OR, cardiac catheterization lab	Total of 489 participants: 160 trauma, 163 emergency department, 67 cardiac catheterization lab, 54 administration, 22 surgery/operative services, 23 medicine and paediatrics; 288 nurses and technicians, 104 physicians, 97 administrative personnel	Trainers from commercial vendor: military and commercial airline pilots proficient in HF engineering, physiology, CRM development, and training	8 h
**Leonard *et al*., 2004**	Clinical projects, site visits, educational sessions, conference calls	Clinical teams from OR, ICU, continuing care (patient transfer), obstetrics, cardiac treadmill unit	Total of 12 clinical teams	Unspecified	3 days
**Helmreich *et al*., 1996**	Simulation with simulator, briefing, self-directed debriefing with videotaped simulation operation	Orderlies, surgical consultants and registrars, anaesthetic consultants and registrars, anaesthetic and surgical nurses	Unspecified	Consultant and senior faculty who received specialized HF training	3 h

CRM, crew resource management; OR, operating room; NOTSS, Non-technical Skills for Surgeons; HF, human factors; PPT, PowerPoint; EMAC, Effective Management of Anaesthetic Crises

**Fig. 2:**
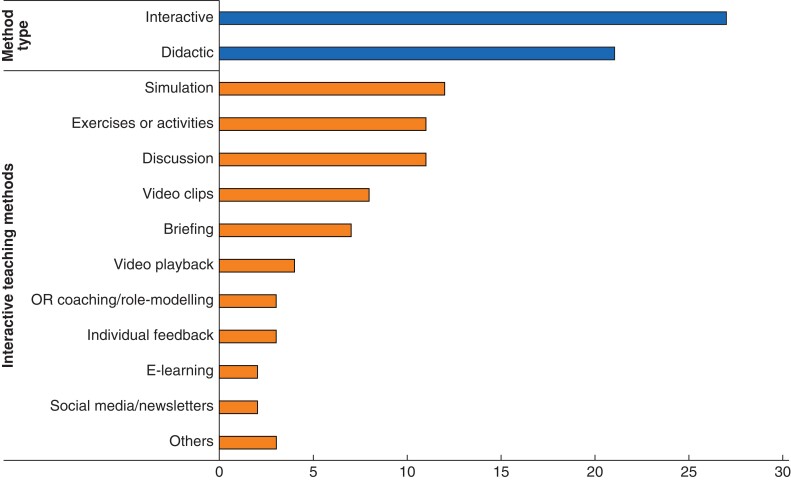
Types of teaching methods used in human factors training interventions OR, operating room.

Six interventions were created in collaboration with a commercial company^22,23,30,32,37–39^, while others were pursued by research groups and experts in HF, CRM, psychology, or other disciplines (*Table 1*). Interactive or non-didactic techniques were applied to teach HF in all 27 interventions, alongside didactic tools such as lectures, presentations, and reading material (*Fig. 2*) in 21 studies. Interactive methods most commonly included simulation (*n* = 12; 44 per cent), group activities or exercises (*n* = 11; 41 per cent), discussion (*n* = 11; 41 per cent), and video clips (*n* = 8; 30 per cent) on patient safety incidents.

### Training content

The key findings reported for each study are listed in *Table 2*. Specific skills and concepts were classified into 226 CIEHF knowledge areas. Of these, 164 (72.6 per cent) were classified under ‘psychology’, 55 (24.3 per cent) under ‘people and systems’, and six (2.6 per cent) under ‘methods and tools’. Only one knowledge area (0.4 per cent) belonged to ‘anatomy and physiology’, while ‘work environment’ was never represented (0 per cent). The inter-rater classification reliability between the two authors was 0.79 (Cohen’s kappa statistic).

**Table 2:** Content and outcomes of human factors training interventions

Study	Skills and concepts taught	Trainee assessment or feedback tool	Key outcomes	Feedback on intervention
**Ansari *et al*., 2020**	Teamwork, situation awareness, communication, decision-making, leadership, conflict resolution, safety culture, cognition, human limitations, stress, handover, briefing/debriefing, task fixation, confirmation bias, transactional analysis, structured communication tools	Hospital Survey of Patient Safety Culture (AHRQ), Kirkpatrick model for training evaluation	Significant improvement in safety culture domains of communication openness, handover, non-punitive response to error, overall safety perception. No change in event reporting	All participants agreed the course was enjoyable and relevant to the work environment. All participants reported that they would recommend the course to a colleague
**Stewart-Parker *et al*., 2017**	Situation awareness, cognitive aids/checklists, communication, communication strategies (SBAR, PACE, closed loop), CRM, leadership, debriefing, fixation error, environmental stressors	NOTECHS for debriefing, self-assessment.	55% increase in confidence for speaking up in difficult situations. 97% of participants continued using their skills after training. Participants reported that the course had helped prevent errors and improve patient safety	All participants reported that the course had a clear structure and explicit objectives. 95% felt that scenarios had good or excellent relevance to clinical practice
**Mancuso *et al*., 2016**	Communication, teamwork, critical language communication, briefing, CRM	CRM observation tool	Significant increase in quantity and quality of communication The increase in quantity was greater in obstetric staff than neonatal staff	Unspecified
**Saleh *et al*., 2016**	Teamwork, behaviours, situation awareness, decision-making, communication, task management, leadership, time/resource management, coping under pressure	NOTSS, NOTECHS, ANTS, OTAS	NOTSS and ANTS had the highest inter-tool and inter-rater consistency, respectively	Participants found the intervention realistic, relevant, and useful
**Stephens *et al*., 2016**	Teamwork, communication, back-up behaviours, leadership, situation awareness, safety culture, briefing, debriefing, incident reporting	Questionnaire for feedback and self-assessed learning	Increased understanding and confidence to enact processes and behaviours supporting safety	Feedback very positive Participants valued working with other specialties away from normal work pressure
**Heaton *et al*., 2016**	Patient safety, teamwork, situation awareness, decision-making, communication, leadership	Questionnaire on non-technical skills, questionnaire for course evaluation	Understanding of non-technical skills improved significantly All participants reported that the perceived importance of these skills was good and very good	All participants enjoyed the course All participants agreed that the course achieved its aims Most participants agreed that the course would improve their clinical practice
**Tsuburaya *et al*., 2016**	Communication, situation awareness, teamwork, leadership, decision-making, coordination, cooperation, monitoring	Written test, Japanese NOTSS, OTAS	Significant improvement in understanding HF and NOTSS system Significant improvement in OTAS scores No differences in NOTSS score but slight improvement in teamwork/communication and leadership	Participants reported that their new visions and skills could be used practically in real clinical scenarios
**Chan *et al*., 2016**	Leadership, teamwork, interpersonal skills, communication, communication strategies (closed-loop, SBAR), assertiveness (five-step assertion model), situation awareness, CRM	Human Factors Attitude Survey, questionnaire for training evaluation	Nurses had significant attitude shifts based on the survey compared to doctors after training Overall positive effect on frontline healthcare professionals’ attitudes	Participants generally found the training useful, relevant, and interesting
**Maertens *et al*., 2016**	Communication, coordination, cooperation, leadership, situation awareness, back-up behaviour	Multiple-choice questions, including evaluation on HF	Vascular surgeons scored higher on multiple-choice questions than students, confirming construct validity	Unspecified
**Timmons *et al*., 2015**	Team performance, patient safety, error reporting/analysis, structured observation, briefing, debriefing, feedback skills, situation awareness, communication, emotional intelligence, teamwork, leadership, stress management, decision-making, change management	Focus groups, semi-structured interviews	Differences related to the status and roles of participants were noted between the emergency department and OR Senior staff better integrated HF into their roles HF is seen as essential to roles at all levels and considered to be part of professional self-regulation	Positive programme evaluation is thought to be acceptable and relevant Staff found it more difficult to implement what was learned to their clinical areas due to informal organizational structures and cultures, especially if involving additional work
**Jones *et al*., 2014**	Situation awareness, decision-making, communication, teamwork, leadership	Advocacy and inquiry approach for a formal critique of performances, self-assessment of confidence in NOTSS skills, online feedback questionnaire	A significant difference between self-assessed confidence in using non-technical skills before and after the course	Participants perceived that training would change their practice and that the skills are transferable to their day-to-day clinical work
**De Korne *et al*., 2014**	Communication, management skills, CRM, patient safety, teamwork, situation awareness, decision-making, personality, unsafe behaviour, leadership, accountability, failure/errors, information processing	Semi-structured interviews to assess safety culture, unstructured observations of trainees	Participants became increasingly aware of safety issues while transitioning from a functionally oriented to a team-oriented culture The number of reported near-incidents increased while the number of wrong-side surgeries stabilized to a minimum	Participants respected aviation expert trainers as role models due to their non-hierarchical external perspective and focused on medical–technical issues
**Davies *et al*., 2014**	Situation awareness, decision-making, communication, teamwork, task management, leadership, use of NOTSS	Questionnaire on HF, questionnaire on effectiveness of training	Participants reported more familiarity with terminology and concepts of HF Participants reported that they would actively change their approach to teamwork and communication	Evaluations were positive overall All participants felt they needed more instruction on the use of observation tools
**Bleakley *et al*., 2006, 2012**	Teamwork, patient safety, communication, leadership, situation awareness, collaboration, briefing, debriefing, close-call reporting	Teamwork Climate in Safety Attitudes Questionnaire	Positive, unidirectional changes in attitudes toward teamwork Participants’ valuing of teamwork activity was improved and sustained	Unspecified
**Hull *et al*., 2012**	Patient safety research, safety culture, communication, teamwork, teamwork assessment (OTAS)	Multiple-choice questions, patient safety survey, OTAS, global course evaluation	Knowledge of surgical patient safety improved significantly Participant confidence and understanding of methodologies to assess OR patient safety and teamwork improved significantly	The workshop was thought to be practical and enhanced understanding of patient safety concepts Some participants commented that training impact would have been even better if delivered in their native language
**Morgan *et al*., 2011**	Communication, task delegation, task management, situation awareness, decision-making, teamwork, behaviours, human errors	ANTS	Overall, ANTS scores improved by 5% with simulations, but debriefing had no effect The ANTS category ‘situation awareness’ improved with debriefing	Unspecified
**Catchpole *et al*., 2010**	Teamwork, briefing, debriefing, time-out, checklists	Teamwork scoring using Oxford NOTECHS	Significant increase in briefing, time-outs, debriefing Intraoperative teamwork has not unequivocally improved	Training well received in general Some perceived training as remedial and inherently critical of frontline staff, especially when persistent systemic issues were not addressed
**Hurlbert and Garrett, 2009**	Situation awareness, communication, teamwork, patient safety, safety culture, briefing	AHRQ survey	Increased number of surgeons using briefings Positive difference in ORs that had a preoperative briefing OR felt less hostile with more briefings As more surgeons did briefings, staff felt that there was more teamwork and openness	Unspecified
**Mason *et al*., 2009**	Decision-making, intuition, cognitive errors, bias, mental imagery, psychomotor skills, situation awareness, personality	The questionnaire, focus group discussions	Decision-making rated as having the most considerable impact on performance The increased perception that work stress and interpersonal difficulties can affect performance Three themes (personal, professional development, trainee–trainer relationship, changing perspective) emerged from the focus group	Views of the course were favourable Integration of aviation concepts was thought to be useful Suggestions included the need for more interactive, scenario-based sessions and focused on the theory–practice gap
**Koutanji *et al*., 2008**	Safety, teamwork, briefing, checklists, communication, situation awareness, leadership, management, decision-making, human error, CRM	Modified NOTECHS (HFRS-M), Safety Climate Survey, Briefing Attitudes Questionnaire, Participant Evaluation of Training Questionnaire for course evaluation	Some attitudes toward briefing improved after training Compared to other trainees, surgeons’ decision-making skill was rated lower than other non-technical skills Overall non-technical skills scores with surgeons were lower than in other professions Training did not significantly improve non-technical skill performance	Overall assessment of simulation scenarios for training was positive
**Marshall and Manus, 2007**	Teamwork, communication, communication strategies (SBAR), Evaluation of communication, behaviours, briefing, debriefing, assertiveness, situation awareness, CRM	Hospital Survey on Patient Safety Culture	7.4% gain on average in 12 dimensions of the patient safety survey post-programme implementation	Participants ranked the training sessions in the 90th percentile in relation to other sessions they had attended
**Undre *et al*., 2007**	Teamwork, safety, crisis management, leadership, communication, decision-making, situation awareness	Modified NOTECHS (HFRS-MS, HFRS-MN, HFRS-MA, HFRS-MO) Participant Evaluation of Training Questionnaire for course evaluation	Scores in leadership and decision-making were lower than communication, team skills, vigilance Surgeons scored lower than nurses on communication and teamwork Surgeons and anaesthetists scored lower than nurses on leadership	Participants assessed the training favourably
**Moorthy *et al*., 2006**	Communication, situation awareness, teamwork, leadership, management skills, time management, resource utilization, assertiveness, decision-making	Modified NOTECHS, participant Evaluation of Training Questionnaire for course evaluation	Variations present within both senior and junior trainees for team skills No differences in HF skills between senior trainees and junior trainees	The majority of participants found the simulation intervention realistic and suitable for team skills training
**Weller *et al*., 2005**	Behaviours, leadership, teamwork, psychology, human performance, crisis prevention, crisis management, production pressure, systems thinking, patient safety	Formative trainee assessment, observation by external evaluators, questionnaire for course evaluation	Most participants reported having mastered the content at a level closer to mastery than beginners	Learning was found to be relevant to practice The course was thought to be appropriate for all levels of training
**Grogan *et al*., 2004**	Behaviours, fatigue management, sleep physiology, cross-checking, communication, decision-making, performance feedback, teamwork, situation awareness, assertiveness, briefing, debriefing, CRM	End-of-course critique, Human Factors Attitude Survey	Positive impact on attitudes towards leadership, coordination, communication, teamwork, recognizing red flags, briefing, debriefing	95% agreed that CRM training would reduce errors in practice Some participants expressed reservations on whether CRM training would transform work practices
**Leonard *et al*., 2004**	Behaviours, safety, communication, communication tools (SBAR), teamwork	Safety Attitude Questionnaire	Led to use of SBAR in perinatal safety, use of checklist and briefing, use of perioperative briefing in surgery	Unspecified
**Helmreich *et al*., 1996**	Teamwork, instruction techniques, briefing, performance feedback	Rating for simulation evaluation	Unspecified	Simulation training rated very highly

AHRQ, Agency for Healthcare Research and Quality; SBAR, Situation, Background, Assessment, Recommendation; PACE, Probe, Alert, Challenge, Emergency; CRM, crew resource management; NOTECHS, Oxford Non-technical Skills; NOTSS, Non-technical Skills for Surgeons; ANTS, Anaesthetists’ Non-Technical Skills; OTAS, Observational Teamwork Assessment for Surgery; HF, human factors; HFRS-M, Human Factors Rating Scale–Modified for Surgeons; HFRS-MN, Nurses; HFRS-MA, Anesthetists; HFRS-MO, Operating Department Practitioners

#### Psychology

**Fig. 3:**
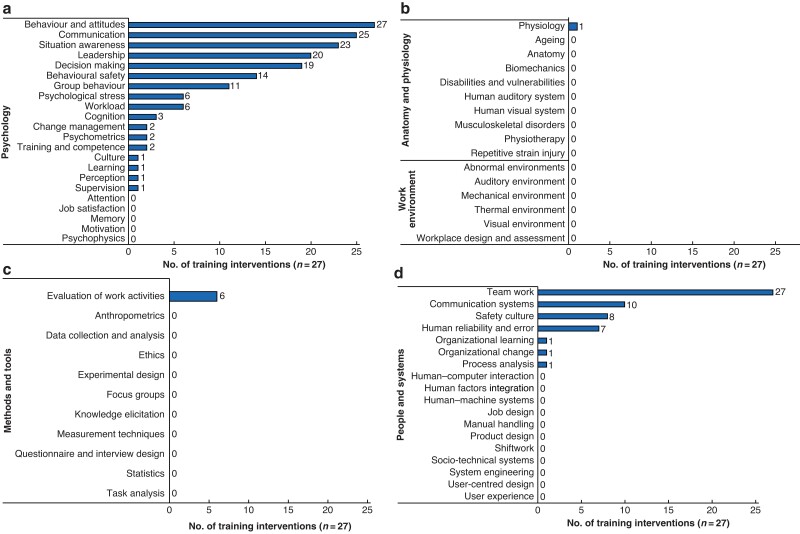
Prevalence of Chartered Institute of Ergonomics and Human Factors knowledge areas based on the content of human factors training interventions **a** Psychology. **b** Work environment and anatomy and physiology. **c** Methods and tools. **d** People and systems.

All 27 interventions included skills or concepts under the knowledge area ‘behaviour and attitudes’ (*Fig. 3a*), often in the context of OR performance and patient safety. Such behaviours and attitudes included communication (*n* = 25; 93 per cent), situation awareness (*n* = 23; 85 per cent), leadership (*n* = 20;74 per cent), and decision making (*n* = 19; 70 per cent). These skills were commonly delivered as part of CRM^26,32,33,37,39–43^, and mostly assessed through behavioural marker systems, such as the Oxford Non-Technical Skills (NOTECHS)^24–27,33,44^, Non-technical Skills for Surgeons (NOTSS)^27,28,31^, and Observational Teamwork Assessment for Surgery (OTAS)^27,28,45^. Psychological stress (*n* = 6; 22 per cent) and workload (*n* = 6; 22 per cent) were also reported around burnout, stress management, and working under pressure^24,27,34^.

#### People and systems

Team work was taught by all 27 interventions (*Fig. 3d*). Communication systems such as SBAR (Situation, Background, Assessment, Recommendation), PACE (Probe, Alert, Challenge, Emergency), briefing strategies, and closed-loop communication were included in 10 studies^22,23,25,30,32,33,36,38–40,46^. By contrast, interactions with equipment and technology (e.g. human computer interaction, human machine systems, or systems engineering) have never been represented.

#### Methods and tools

Only the knowledge area of ‘evaluation of work activities’ (*n* = 6; 22 per cent), was represented under methods and tools (*Fig. 3c*). This included teaching strategies for structured observation and feedback, work evaluation in the context of research, analysis of errors, and evaluation of non-technical skills^34,37,39,45,47,48^. Knowledge areas related to research techniques (e.g. data collection and analysis, experimental design, focus groups, and questionnaire and interview design) were not included in any HF teaching.

#### Anatomy and physiology

The knowledge area of physiology was represented in one training offer (*Fig. 3b*)^37^. The study encompassed lessons around sleep physiology and the effects of sleep disruptions on performances.

#### Work environment

No HF training offers included concepts related to audiovisual, thermal, and mechanical interactions, environmental distractions such as noise pollution, or the workplace design and assessment, including OR design and ergonomics.

### Training outcomes and feedback

All studies recording participants’ feedback reported positive responses to the overall training or its components^24–28,30,31,33–40,42–45,47–49^. In some studies, participants perceived their training as realistic or practice-changing^24,27,28,31,37,42^. Adding technical skills to the training^27^, incorporating interdisciplinary learning^47^, and integrating experts in HF training^43,49^, was recognized to improve learning.

The types of outcomes reported varied between studies (*Table 2*). Several training interventions demonstrated an improved attitude towards or quantity of briefings or time-outs^26,37,40^. Assessment of non-technical skills like teamwork and communication were variable. One study described lower communication, team work performance and attitude, decision-making, and leadership scores in surgeons *versus* nurses, while another demonstrated lower overall non-technical skills scores in surgeons *versus* other professions^25,26^. When attitudes or awareness around safety were assessed, improvements were seen with training^22,23,26,30,33,34,37,39,40,42,43,45,47^.

Of the studies that assessed behavioural marker systems or evaluation methods in the context of HF training, Saleh *et al.* reported high inter-tool and inter-rater consistency with NOTSS and ANTS^27^. Tsuburaya *et al*. also demonstrated feasibility in using a Japanese version of NOTSS (jNOTSS) and OTAS^28^. In another study, construct validity for assessing and scoring HF skills within a larger endovascular training programme was demonstrated^29^.

Some of the challenges faced during the HF training included criticisms of frontline staff inherent to the intervention^44^, resistance to changes^34,48^, gaps between theory and practice, doubts on the actual effect of the intervention^37,38,49^, and a sense of loss of autonomy^38,39^. Suggested solutions to overcome these barriers included building organizational commitment or culture around HF goals^22,23,44^, inclusion of stakeholders at all levels^34,44^, encouraging physician and nursing leadership^36,38,39^, enhancing authenticity in HF initiatives by reserving time, funding, and resources^34^, teaching through more interactive methods^49^, and providing continuous or multiple training sessions rather than a single intervention^37,43^.

## Discussion

A significant amount of research has been undertaken to examine the elements of the OR that produce high-reliability systems^4,5^. These elements have often been focused on well-established fields of HF used in other high-stakes environments and thus progressively extended for the assessment of safety in the OR^4,6,50^.

This review demonstrated that HF training for the operative setting predominantly focuses on teaching interpersonal behaviours related to patient safety, approximating the emerging literature around non-technical skills in surgery^51,52^. Skills related to teamwork, communication, situation awareness, decision making, and leadership have been shown to impact performance in the OR^3,53–55^, and consequently, have been incorporated into training models with behavioural rating systems like NOTSS or NOTECHS, aimed at individual and team assessment and teaching^51–53,56^.

However, striving for a high-reliability organization entails more than just optimizing human non-technical skills^9,57^. Analysis of flow disruptions in surgery has uncovered other factors, such as equipment and technology problems, resource accessibility issues, and suboptimal systems organization, all leading to patient safety incidents^5^. Interestingly, these areas of knowledge were not represented by any of the included studies.

Likewise, providers’ skills and experience may go beyond individual competencies. While psychological stress and workload have been emphasized by several studies to ultimately affect performance in the OR^24,27,30,34,35,37,41,49^, recent literature has shifted focus from only individual resilience to all contributors to providers burnout, including suboptimal usability of technology, poor funding arrangements, staffing shortage, and workflow interruptions^58^. However, these knowledge areas were not applied in any of the included interventions, suggesting that HF applied to the operative setting probably has still not addressed the full range of sociotechnical factors affecting providers’ experience and potentially influencing their response to OR crisis. Unlike other high-stakes environments, it is less likely that elements beyond individual behaviours and skills will be used in these situations to anticipate and control OR threats, suggesting that only behavioural changes without considering systems and environmental factors are limited strategies^5,50,59,60^.

The content of HF training was reflected by the training delivery method in the included studies. Simulation-based learning has been found to develop sustainable teamwork behaviours that cannot be consistently practised and demonstrated *in vivo*, making it an ideal tool for teaching non-technical skills.^61^ The use of video clips of intraoperative recordings was also frequently applied as a review and debrief method^26,27,36,41,43^, suggesting that capturing provider behaviours in ‘naturalistic settings’ is crucial for standardized and realistic approaches in HF education.

This study has several limitations. Firstly, searching with different databases, keywords, and languages may have identified additional research. However, the chosen databases had broad coverage of the healthcare literature, confirming the completeness of the current search. Secondly, a grey literature search was not conducted and may eventually require a separate study to examine the curriculum objectives of different faculties, institutions, and HF training companies. It is also important to recognize that skills and concepts of HF training interventions may be taught elsewhere under separate labels. Lastly, the inter-rater reliability for the classification of CIEHF knowledge areas demonstrated some, albeit few, disagreements between the two authors. All knowledge areas were reviewed, and disagreements were resolved by consensus to ensure a consistent and accurate approach.

HF investment and education can ultimately facilitate the integration of a shared culture that supports safety initiatives for the operative environment. In particular, shifting the focus from individual traits to the interchange between work practices and provider behaviours can raise awareness of how safety incidents occur^58^. The operative context requires the integration of specific concepts and skills and relevant knowledge from established HF industries. The recruitment of HF experts can facilitate this process by providing an external perspective beyond the OR hierarchy^43^. Although implementing HF requires a significant investment of resources and funding, HF training should be longstanding to create a more longitudinal impact^10,13^. HF integration may eventually lead to a considerable return on investments as high as 7:1, limiting costly safety incidents^62^. As research evolves and introduces new dynamic sociotechnical factors (e.g. novel technologies and new healthcare roles), HF education for the operative space must adapt to expand the range and scope of HF for the operating room.

## Supplementary Material

zrac011_Supplementary_Data

## Data accessibility

The data that support the findings of this study are available from the corresponding author upon reasonable request.
