# Supplementary material for: Exploring human factors in the operating room: scoping review of training offerings for healthcare professionals
Source: BJS Open. 2022 Mar 29;6(2):zrac011. doi: 10.1093/bjsopen/zrac011 (PMC8963294; doi:10.1093/bjsopen/zrac011)
Supplement: zrac011_Supplementary_Data [file zrac011_supplementary_data.zip › Supplementary_material.docx]

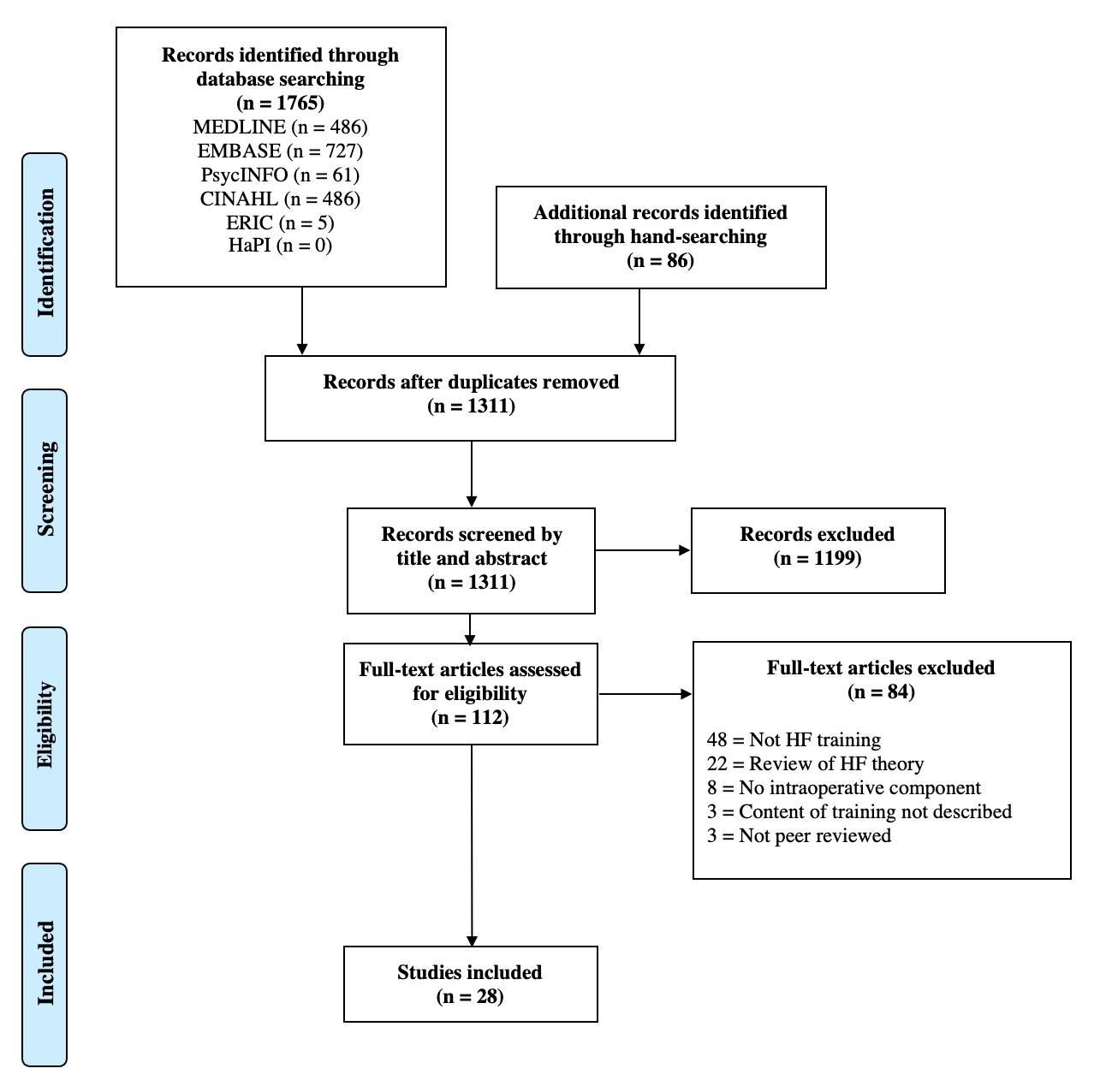


**Figure S1. PRISMA flow diagram of the literature search and screening.**

**Table S1. MEDLINE Literature Search Strategy**

|  | **Search Terms** | **Results** |
| --- | --- | --- |
| 1 | human factor*.mp. | 7379 |
| 2 | Operating Rooms/ | 13814 |
| 3 | exp Intraoperative Period/ | 28116 |
| 4 | Intraoperative Care/ | 16923 |
| 5 | exp Monitoring, Intraoperative/ | 19995 |
| 6 | exp Specialties, Surgical/ | 198817 |
| 7 | (operating room* or operating theatre* or operating theater* or operating table*).mp. | 45940 |
| 8 | (intraoperative or intra-operative).mp. | 179012 |
| 9 | surg*.mp. | 3147513 |
| 10 | 2 or 3 or 4 or 5 or 6 or 7 or 8 or 9 | 3273574 |
| 11 | 1 and 10 | 764 |
| 12 | exp Education/ | 807531 |
| 13 | exp Learning/ | 384251 |
| 14 | exp Surgeons/ed [Education] | 936 |
| 15 | exp Anesthetists/ed [Education] | 798 |
| 16 | exp Nurses/ed [Education] | 8377 |
| 17 | exp Nurse Specialists/ed [Education] | 3921 |
| 18 | exp Nursing Staff/ed [Education] | 14450 |
| 19 | (train* or educat* or teach* or learn* or intervention? or curricul* or program* or course* or coach* or workshop* or lecture* or class* or lesson* or mentor*).mp. | 5396331 |
| 20 | (drill* or exercise* or school* or instruction* or apprentice*).mp. | 774516 |
| 21 | 12 or 13 or 14 or 15 or 16 or 17 or 18 or 19 or 20 | 5984948 |
| 22 | 11 and 21 | 486 |

**Table S2. CIEHF Knowledge Areas of Human Factors and Ergonomics^a^**

| **Anatomy and Physiology** | **Psychology** | **People and Systems** | **Work Environment** | **Methods and Tools** |
| --- | --- | --- | --- | --- |
| Ageing | Attention | Communication systems | Abnormal environments | Anthropometrics |
| Anatomy | Behaviour and attitudes | Human computer interaction | Auditory environment | Data collection and analysis |
| Biomechanics | Behavioural safety | Human factors integration | Mechanical environment | Ethics |
| Disabilities and vulnerabilities | Change management | Human machine systems | Thermal environment | Evaluation of work activities |
| Human auditory system | Cognition | Human reliability and error | Visual environment | Experimental design |
| Human visual system | Communication | Job design | Workplace design and assessment | Focus groups |
| Musculoskeletal disorders | Culture | Manual handling |  | Knowledge elicitation |
| Physiology | Decision making | Organisational change |  | Measurement techniques |
| Physiotherapy | Group behaviour | Organisational learning |  | Questionnaire and interview design |
| Repetitive strain injuries | Job satisfaction | Product design |  | Statistics |
|  | Leadership | Process analysis |  | Task analysis |
|  | Learning | Safety culture |  |  |
|  | Memory | Shiftwork |  |  |
|  | Motivation | Socio-technical systems |  |  |
|  | Perception | System engineering |  |  |
|  | Psychological stress | Team work |  |  |
|  | Psychometrics | User centred design |  |  |
|  | Psychophysics | User experience |  |  |
|  | Situation  awareness |  |  |  |
|  | Supervision |  |  |  |
|  | Training and competence |  |  |  |
|  | Workload |  |  |  |

^a^ Definitions of each knowledge area can be found on the CIEHF website.

**Table S3. Characteristics of Included HF Training Studies**

| **Study** | **Country** | **Keywords Reported** | **Study and Intervention Design** | **Summary of Overall Intervention** | **MERSQI Score** |
| --- | --- | --- | --- | --- | --- |
| Ansari et al. 2019 | United Kingdom | Human factors, Patient safety, Training, Maternity | Observational, prospective, pre-post study design; Pilot intervention | Maternity-oriented HF on safety culture for maternity unit staff | 11 |
| Stewart-Parker et al. 2017 | United Kingdom | Nontechnical skills, Human factors, Multiprofessional training, Simulation, Patient safety | Multi-methods, pre-post study design; Pilot intervention | Nontechnical skills and HF course for OR staff | 9.5 |
| Mancuso et al. 2016 | United States of America | Cesarean births, Communication, Human factor training, Obstetrics | Prospective, pre-post study design; Mandatory intervention | CRM training for obstetric and neonatal teams | 13 |
| Saleh et al. 2016 | United Kingdom | None | Prospective, multi-methods study design; Pilot intervention | Immersive simulation HF training for ophthalmic surgical teams | 14.5 |
| Stephens et al. 2016 | United Kingdom | Interprofessional collaboration, Interprofessional learning, Patient safety, Perioperative, Team culture, Team learning | Quasi-experimental post-test study design; Elective/ optional intervention | Interprofessional training course focused on crises and HF for perioperative practitioners | 10 |
| Heaton et al. 2016 | United Kingdom | Teaching, Learning, Simulation, Non-technical, Surgery | Multi-methods, pre-post study design; Pilot intervention | Simulated nontechnical skills course for orthopedic trainees | 8.5 |
| Tsuburaya et al. 2016 | Japan | Operating room, Non-technical skills, Human factors, Surgery | Pre-post study design; Pilot intervention | Nontechnical skills e-learning for upper gastrointestinal surgeons | 14.5 |
| Chan et al. 2016 | Hong Kong | Patient safety, Crew resource management, CRM, Healthcare, Teamwork | Multi-method, pre-post study design; Elective/ optional intervention | CRM classroom-based training for healthcare professionals | 10.5 |
| Maertens et al. 2016 | Belgium | Medical knowledge, Practice-based learning, Interpersonal and communication skills, Surgical training, Proficiency based, Endovascular intervention, Assessment | Non-equivalent groups study design; Pilot intervention | Endovascular skills training for medical students and vascular surgeons | 14 |
| Timmons et al. 2015 | United Kingdom | Anaesthesia, Education, Teaching, Emergency department | Qualitative, longitudinal study design; Pilot intervention | Aviation-derived HF training for emergency room and OR staff | NA |
| Jones et al. 2014 | United Kingdom | None | Multi-method, pre-post study design; Mandatory intervention | HF training course for surgical trainees | 14.5 |
| De Korne et al. 2014 | Netherlands | Quality improvement, Professional education, Hospitals, Teamwork | Mixed-methods, pre-post study design; Pilot intervention | Aviation-based team resource management program for healthcare professionals | 13.5 |
| Davies et al. 2014 | Australia | None | Descriptive article; Pilot intervention | HF training workshops for OR staff | NA |
| Bleakley et al. 2006, 2012 | United Kingdom | Interprofessional working, Operating theatre, Collaborative inquiry, Teamwork, Self-sustaining educational intervention | Mixed-methods, pre-post, interrupted time series study design; Mandatory intervention | Teamwork education for OR personnel | 9.5 |
| Hull et al. 2012 | Colombia | Patient safety, Teamwork, Operating room, Human factors | Mixed-methods, pre-post study design; Pilot intervention | Surgical safety research training program | 12 |
| Morgan et al. 2011 | Canada | High-fidelity simulation, Debriefing, Human factors, Performance | Evaluation of previous randomized controlled trial; Pilot intervention | High-fidelity simulation debriefing training for anesthesiologists | 14.5 |
| Catchpole et al. 2010 | United Kingdom | Teamwork, Human factors, Training, Intervention, Safety | Prospective, mixed-methods, pre-post design; Pilot intervention | Multicentre aviation-style HF training for surgical teams | 12.5 |
| Hurlbert and Garrett 2009 | United States of America | Operating room, Airline industry, Room staff, Operating room staff | Multi-methods study design; Elective/ optional intervention | HF training for OR staff | 10 |
| Mason et al. 2009 | United Kingdom | Human factors, Surgical performance, Training, Evaluation, Qualitative | Observational, mixed-methods, pre-post study design; Pilot intervention | Psychological skills training course for surgeons | 9.5 |
| Koutantji et al. 2008 | United Kingdom | Team training, Crew resource management, Mixed model ANOVAs, Theatre team, Crew resource management training | Multi-methods, pre-post study design; Pilot intervention | Surgical teamwork and safety training using briefings in simulated crisis scenarios | 13 |
| Marshall and Manus 2007 | United States of America | None | Multi-method, pre-post study design; Elective/ optional intervention | HF program based on CRM training for OR staff | 8.5 |
| Undre et al. 2007 | United Kingdom | None | Observational, cross-sectional study design; Pilot intervention | Simulated crisis scenarios module for OR teams | 13.5 |
| Moorthy et al. 2006 | United Kingdom | None | Assessment of training intervention; Pilot intervention | Non-equivalent groups, posttest design; Surgical crises management skills training for surgical trainees | 13.5 |
| Weller et al. 2005 | Australia, New Zealand | Medical education, Patient simulation, Obstetric anaesthesia, Medical errors, Patient safety | Mixed-methods study design; Pilot intervention | Training for management of anesthetic emergencies for anesthesiologists | NA |
| Grogan et al. 2004 | United States of America | None | Mixed-methods, pre-post study design; Elective/ optional intervention | Aviation-based teamwork training for healthcare professionals | 10.5 |
| Leonard et al. 2004 | United States of America | None | Descriptive article; Mandatory intervention | HF training focused on teamwork and communication | NA |
| Helmreich et al. 1996 | Switzerland | None | Descriptive article; Mandatory intervention | Team-oriented medical simulation training for the OR team | NA |
